# Wallerian-like axonal degeneration in the optic nerve after excitotoxic retinal insult: an ultrastructural study

**DOI:** 10.1186/1471-2202-11-97

**Published:** 2010-08-13

**Authors:** Sarabjit K Saggu, Hiren P Chotaliya, Peter C Blumbergs, Robert J Casson

**Affiliations:** 1 Ophthalmic Research Laboratories, Hanson Institute, and The University of Adelaide, SA 5000, Australia; 2 Centre for Neurological Diseases, Hanson Institute, Adelaide, SA 5000, Australia

## Abstract

**Background:**

Excitotoxicity is involved in the pathogenesis of a number neurodegenerative diseases, and axonopathy is an early feature in several of these disorders. In models of excitotoxicity-associated neurological disease, an excitotoxin delivered to the central nervous system (CNS), could trigger neuronal death not only in the somatodendritic region, but also in the axonal region, via oligodendrocyte N-methyl-D-aspartate (NMDA) receptors. The retina and optic nerve, as approachable regions of the brain, provide a unique anatomical substrate to investigate the "downstream" effect of isolated excitotoxic perikaryal injury on central nervous system (CNS) axons, potentially providing information about the pathogenesis of the axonopathy in clinical neurological disorders.

Herein, we provide ultrastructural information about the retinal ganglion cell (RGC) somata and their axons, both unmyelinated and myelinated, after NMDA-induced retinal injury. Male Sprague-Dawley rats were killed at 0 h, 24 h, 72 h and 7 days after injecting 20 nM NMDA into the vitreous chamber of the left eye (n = 8 in each group). Saline-injected right eyes served as controls. After perfusion fixation, dissection, resin-embedding and staining, ultrathin sections of eyes and proximal (intraorbital) and distal (intracranial) optic nerve segments were evaluated by transmission electron tomography (TEM).

**Results:**

TEM demonstrated features of necrosis in RGCs: mitochondrial and endoplasmic reticulum swelling, disintegration of polyribosomes, rupture of membranous organelle and formation of myelin bodies. Ultrastructural damage in the optic nerve mimicked the changes of Wallerian degeneration; early nodal/paranodal disturbances were followed by the appearance of three major morphological variants: dark degeneration, watery degeneration and demyelination.

**Conclusion:**

NMDA-induced excitotoxic retinal injury causes mainly necrotic RGC somal death with Wallerian-like degeneration of the optic nerve. Since axonal degeneration associated with perikaryal excitotoxic injury is an active, regulated process, it may be amenable to therapeutic intervention.

## Background

Excitotoxicity, the mechanism involved in the pathogenesis of neurological diseases, including stroke, motor neuron disease (MND), Alzheimer's disease (AD), retinal ischemia and glaucoma [1-12], is classically considered as a somatodendritic insult due to prolonged or excessive activation of excitatory amino acid receptors. Studies have also indicated axonopathy as an early feature in neurodegenerative diseases associated with excitotoxicity [13-16]. It is unclear whether the nerve degeneration associated with excitotoxicity is due to primary insult at the perikaryal level in the grey matter or a primary excitotoxic injury in the white matter.

An excitotoxin delivered to the central nervous system (CNS), could trigger injury not only in the somatodendritic region, but simultaneously, in the axonal region. As retinal ganglion cells (RGCs) axons have a relatively long projection within the eye before reaching the optic nerve, intravitreal excitotoxic injury, which is physically isolated from the retro-orbital axons, may be the result of toxic insult to RGCs and/or intraocular axonal compartment. Studies have confirmed perisynaptic localisation of N-methyl-D-aspartate (NMDA) receptors in RGCs [17]. Although there is evidence for the presence of non-NMDA glutaminergic receptors for alpha-amino-3-hydroxy-5-methyl-4-isoxazolepropionic acid (AMPA) and kainite in the postsynaptic myelinated axons in the central neurons [18] and the expression of NMDA receptors on oligodendrocyte processes in white matter [19], there is no direct evidence of presence of functional NMDA receptors on axons [20]. Therefore, retro-orbital optic nerve axonal degeneration observed in NMDA-induced retinal insult is logically a consequence of primary damage to RGCs; however, damage to intraorbital axons can also be considered a primary site of insult, if future studies provide direct evidence for the presence of functional NMDA receptors over axons.

The retina and optic nerve, as approachable regions of the CNS, provide a unique substrate to investigate the effect of NMDA induced excitotoxic RGC injury on the optic nerve axons. We previously noted that NMDA induced retinal injury produced an axonopathy which was synchronous with the somal degeneration of RGCs and which was most prominent in the more distal portions (closer to the midbrain) of the axon [21]. To our knowledge, despite numerous reports about excitotoxic neuronal death in the retina, the "downstream" ultrastructural changes in the optic nerve (the RGC myelinated axons) have never been reported. In the current study, we provide ultrastructural information about the RGC somata and their axons, after NMDA-induced retinal injury.

## Methods

### Experimental model

Male Sprague-Dawley rats (*n *= 8) weighing 300-350 g [Institute of Medical and Veterinary Sciences (IMVS), Adelaide, South Australia] were kept at room temperature, with food and water available ad libitum. Adequate care was taken to minimise pain and discomfort for the animals used in this study and the experiments were conducted in accordance with the Australian and international standards on animal welfare. All experiments were approved and monitored by the IMVS, Animal Ethics Committee (Approval No. 53/06).

The excitotoxic RGC injury model was prepared in a manner similar to that previously described [22,23]. After anaesthetising the rats with isoflurane (2.5 L/min isoflurane in 2.5 L/min oxygen), instilling topical 0.4% benoxinate drops in both eyes and applying a sterile loop around the globes, a single dose of 5 μl of 4 mM NMDA (20 nmol, source- Sigma Aldrich, USA) was injected slowly over 30 seconds into the vitreous space of the left eye using a microsyringe fitted with a 30-gauge needle. Right eyes received 5 μl of the NMDA vehicle (sterile 0.9% saline) to serve as controls.

Sets of animals (n = 8 per group) were killed humanely by cardiac perfusion at various time intervals: immediately, 24 hrs, 72 hrs and 7 days after injection. Under deep anaesthesia, animals were killed by intracardiac perfusion with a solution of 2.5% glutaraldehyde with 4% paraformaldehyde in 0.1 M phosphate buffer pH 7.4. To minimise stretch injury to the optic nerve caused by direct enucleation of the globe, the eye, optic nerves and tracts were dissected via a craniotomy. Eyes were separated by cutting the optic nerve 1 mm behind the globe. The optic nerve was divided into proximal (nearer to the globe) and the distal segment (nearer to the midbrain).

### Tissue preparation

Tissues perfused with 2.5% glutaraldehyde were transferred into polypropylene vials and post-fixed in the same fixative overnight at room temperature. The specimens were then rotator-rinsed in sodium cacodylate buffer (with sucrose, pH 7.4) for 30 minutes, and post-fixed in 1% osmium tetroxide (OsO_4_) overnight at room temperature. The specimens were the re-rinsed in sodium cacodylate buffer three times for 30 minutes each, and then dehydrated in a graded alcohol series (70%, 95% and 100%). Dehydrated tissues were then infiltrated and later embedded in fresh TAAB-Epoxy resin with propylene oxide (2-epoxypropane) used as a clearing agent.

### Sectioning and staining

Polymerised resin blocks containing tissue specimens were trimmed and semi-thin sections (0.5 μm) were cut on a mechanical ultramicrotome using a glass knife. Floating each section onto a water bath, sections were collected on labelled polysine slides, dried on the hot plate for 1 hour and stained with toluidine blue. Finally, sections were mounted and cover-slipped. Ultrathin sections (60-80 nm) were cut in the same manner as semi-thin sections, but using a diamond knife. Sections picked on 150 mesh acetone-washed copper grids and dried overnight were stained with Uranyl acetate and Lead citrate stains.

## Results

### RGCs in control retinas

**Figure 1:**
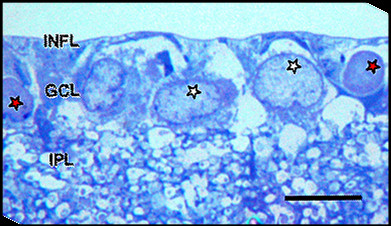
**LM appearance of resin-embedded semi-thin section of saline control normal inner retina stained with Toluidine blue stain**. INFL: inner nerve fibre layer, GCL: ganglion cell layer, IPL: inner plexiform layer. Ganglion cell layer shows prominent nuclei of RGCs (white stars) and amacrine cell (red stars). Bar = 10 μm.

The ultrastructure of retina was interpreted in conjunction with the light microscopy (LM) by an experienced neuropathologist (PCB). In the ganglion cell layer (GCL) of saline injected control eyes, most numerous large-sized cells containing pale nuclei with one or two nucleoli were identified as RGCs, in comparison to amacrine cells, which were smaller and had dark staining nuclei [24,25]. Microglial cells were identified as occasional small-sized cells, with short processes and elongated nuclei. The inner plexiform layer (IPL) showed sections of dendritic processes of RGCs. (Figure 1)

**Figure 2:**
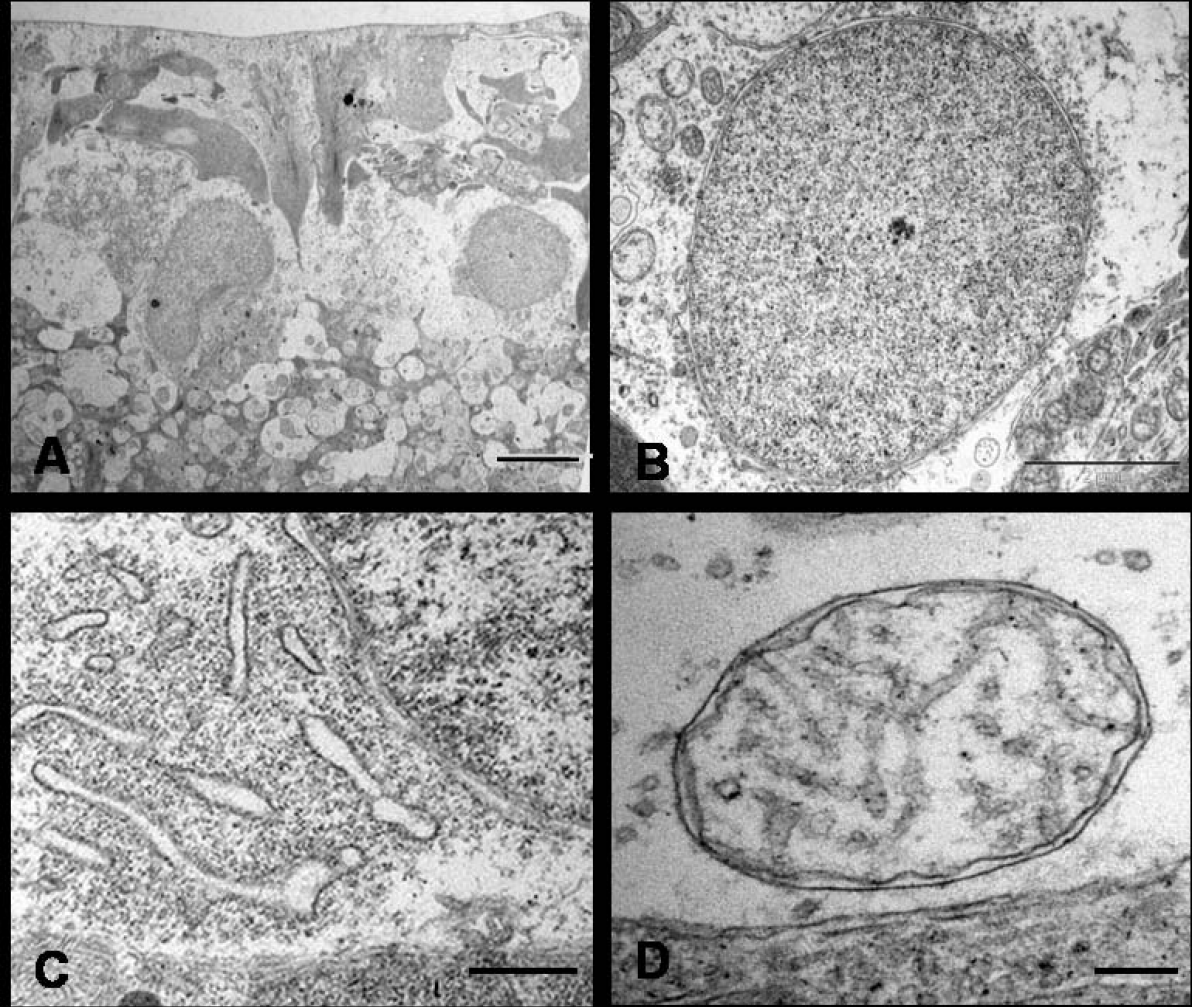
**Ultrastructural appearances of normal RGCs from saline injected eye**. Typical RGCs are seen (A, Bar = 5 μm) with normal nuclei (B, Bar = 2 μm), endoplasmic reticulum (C, Bar = 1.7 μm) and mitochondrion (D, Bar = 20 nm).

Under transmission electron microscopy (TEM), control RGCs had a well-defined continuous plasma membrane, and a non-uniform distribution of organelles in the cytoplasm, with maximum concentration in the perinuclear region. RGCs contained tubular sacs of rough endoplasmic reticulum (rER) surrounded by large numbers of ribosomes (Nissl bodies). Mitochondria were identified as round or oval double- membrane structures with characteristic cristae. In addition, the cytoplasm contained elements of Golgi apparatus (GA), free ribosomes and microtubules sectioned at various angles. A large round nucleus, surrounded by a double layered nuclear membrane, contained homogeneously dispersed karyoplasm (chromatin material) and one or two electron dense nucleoli. RGCs from the control saline-injected eyes showed a similar normal profile at all time points. (Figure 2)

### Ultrastructural changes in RGCs

**Figure 3:**
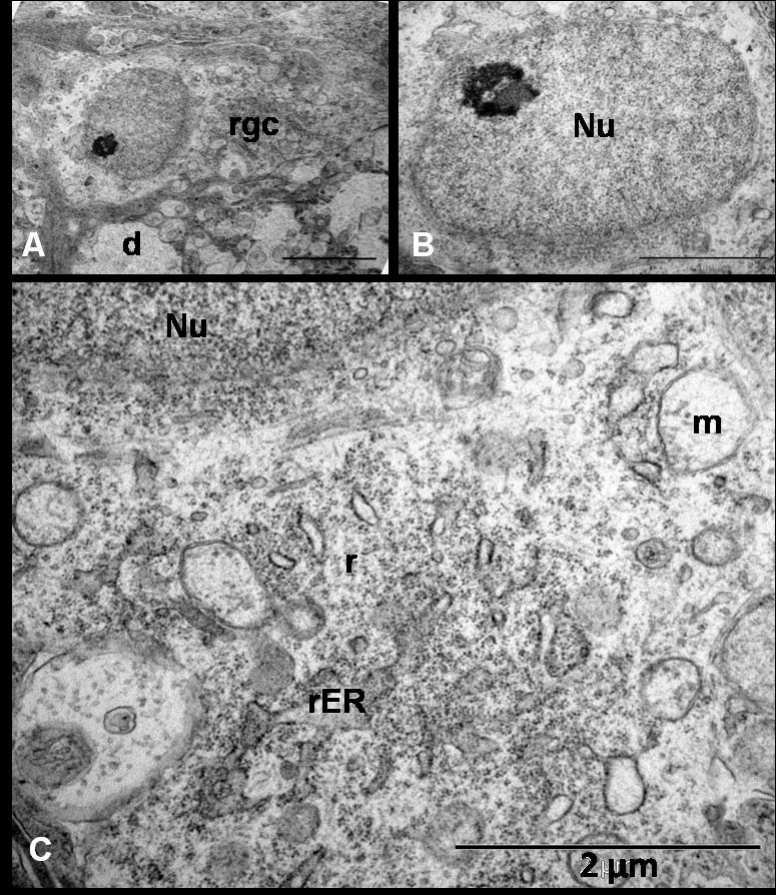
**Ultrastructural appearance of RGCs 24 hrs after NMDA injection shows (A, Bar = 5 μm) early dense appearance of the cell (rgc) cytoplasm with dendritic swelling (d) and normal euchromatic nucleus with a prominent nucleolus (B, Bar = 2 μm)**. Notice the dilatation of mitochondrion (m) and endoplasmic reticulum (ER) as well as some scattered ribosomes (r) in the cytoplasm (C, Bar = 2 μm).

Intravitreal administration of NMDA resulted in excitotoxic damage to RGCs which began as early as 24 hrs. At 24 hrs, 10-20% RGCs showed cytoplasmic engorgement with swelling of numerous dendritic processes giving a spongiform appearance to the IPL. Cytoplasm of swollen RGCs appeared dense and uniformly granular due to scattered ribosomes. Some mitochondria appeared swollen and the rER appeared slightly vacuolated. The cell membrane appeared intact and no nuclear changes were seen at this stage. (Figure 3)

**Figure 4:**
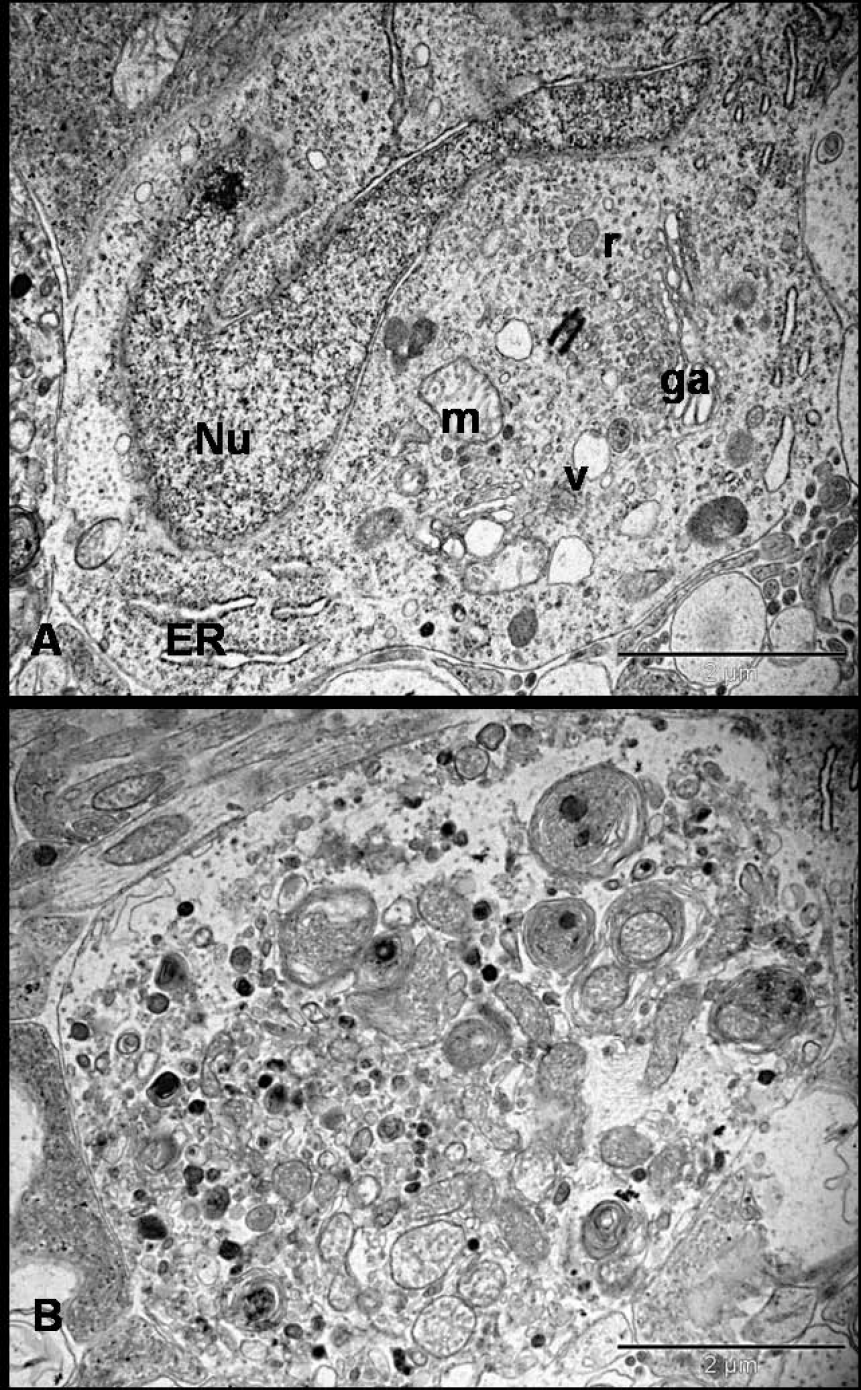
**Ultrastructure appearance of RGCs 72 hrs after NMDA injection**. Neurons are found at different stages of degeneration and the neuronal death is morphologically 'necrotic'. Figure A shows the hyperconvoluted nucleus and cytoplasm filled with multiple vacuoles, ribosomes and swollen organelle. Figure B shows severe necrotic cell death where the cell has lost its architecture and converted into debris. There is no demarcation between cytoplasm and electron-dense bodies. The cytoplasm is studded with the multi-laminated myelin figures and damaged organelles (Bars = 2 μm); Nu = nucleus, ER = endoplasmic reticulum, ga = golgi apparatus, m = mitochondria, v = vesicles, r = ribosomes.

At 72 hrs, the dendritic swelling persisted and the ganglion cell density decreased. RGCs displayed a necrotic form of cell death with features of degeneration seen in a continuum of changes. Most abnormal RGCs had an intact cell membrane with their cytoplasmic matrix containing free monomeric ribosomes, vesicles and dilated cisterns of ER as well as GA. In conjunction with the disaggregation of polyribosomes and disintegration of vacuolated ER, most mitochondria were irregularly oedematous. Some morphologically normal mitochondria were still evident in affected cells. Some cells showed early features of nuclear damage, such as hyperconvoluted nuclei and chromatin condensation into small clumps abutting the nuclear envelope. Nucleoli, however, were morphologically normal. Some RGCs displayed extreme cytolysis and loss of architecture in the form of disrupted cytoplasmic organelles. Their nuclear envelope and organelle membranes were fragmented. Damage was so severe that demarcation between nucleus and cytoplasm was impossible in some cells. Electron-dense clumped nuclear remnants were dispersed into the cytoplasm, which contained vacuolated and rupturing organelles and onion-like multi-laminated 'myelin figures'. (Figure 4)

**Figure 5:**
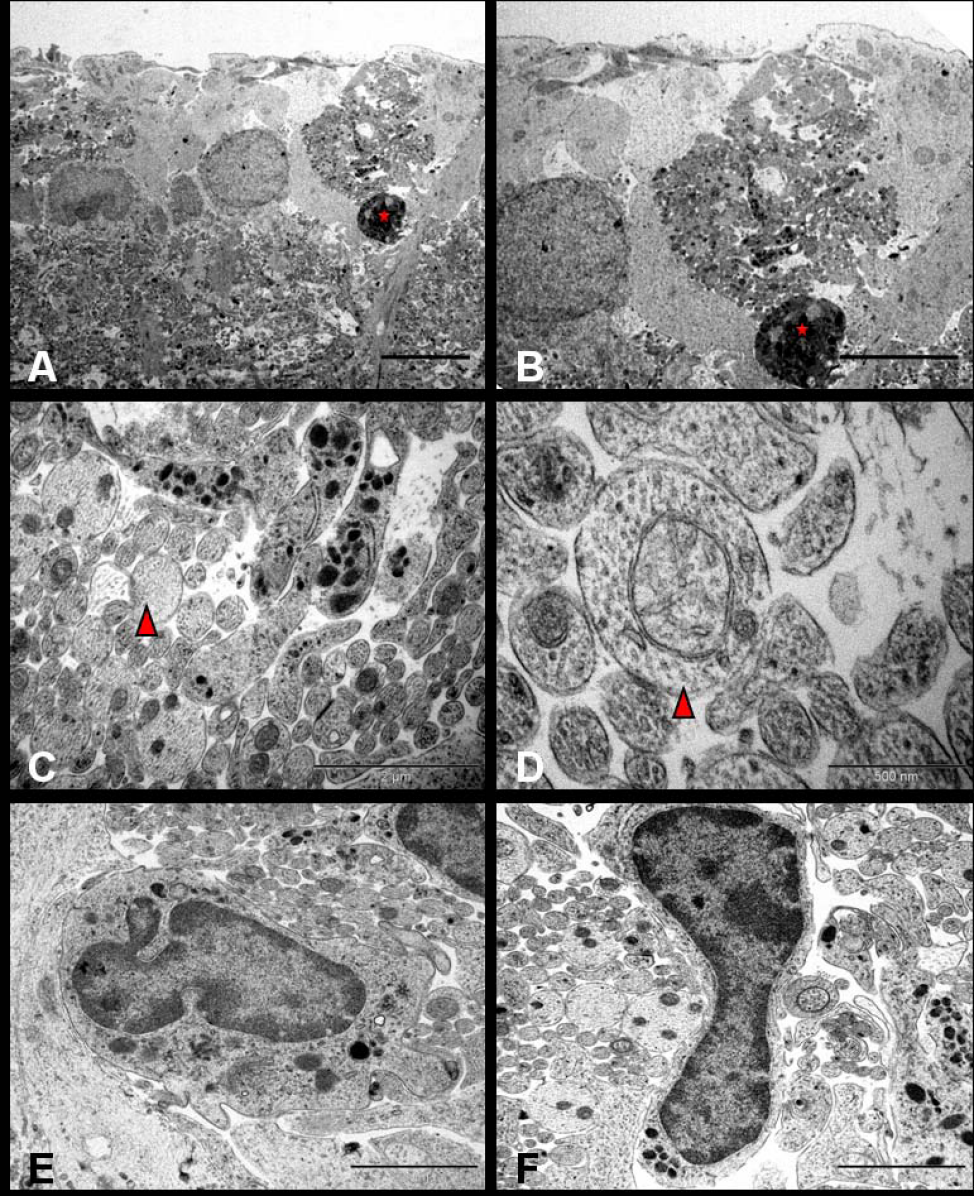
NMDA-induced ultrastructural changes in RGCs at 7 days where necrotic cell in the form of highly electron-dense neuronal debris (Red star, A & B, Bar = 2 μm) is seen lying adjacent to numerous membrane-bound microtubule-rich neuritic processes (C, red triangle, Bar = 2 μm) identified as dendrites under high power (D, red triangle, Bar = 500 nm); Figure E and F show reactive microglia surrounding the dendritic sprouts. (Bars = 2 μm).

At 7 days, the IPL appeared markedly thinned, with dendrites becoming shrunken and dense. As the purpose of this study was to explore ultrastructural changes, no quantification of IPL thickness was attempted here. However, statistical analysis of retinal thinning was done using light microscopy and results published previously [21]. The GCL showed sparse distribution of RGCs, but preservation of most amacrine cells. At this time point, damaged RGCs showed electron-dense neuronal debris remaining in contact with clusters of reactive microglial cells and astrocytic processes. Numerous dendritic processes, recognised by their higher microtubule composition in comparison to axons, were seen distributed in the GCL. These dendrites were packed in the form of clusters, which occupied the empty spaces created by necrotic RGCs. Compared to the dendrites in the IPL, these processes appeared normal in terms of filamentous and organelle composition, with many displaying mitochondria of normal morphology. (Figure 5)

### Ultrastructure of normal optic nerve

**Figure 6:**
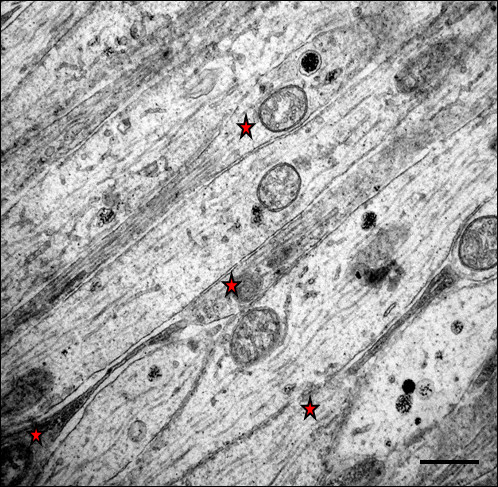
**TEM of the parallel running longitudinal sections of the intraorbital axons in saline injected control retina immediately after the injection**. A few fibres cut to a substantial length showed varicosities (red star) and intervaricosity regions simultaneously (Bar = 1 μm).

The parallel-cut intraocular portion of optic nerve axons seen in retinal sections from saline injected control eyes showed 0.25 to 1 μm thick unmyelinated axons running longitudinally in the INFL with axoplasm showing uniformly distributed longitudinal cytoskeletal filaments and organelles. In some axons with a substantial length of axon visible, mitochondria-rich varicosities were separated by narrowed portion of filament rich axons. (Figure 6)

**Figure 7:**
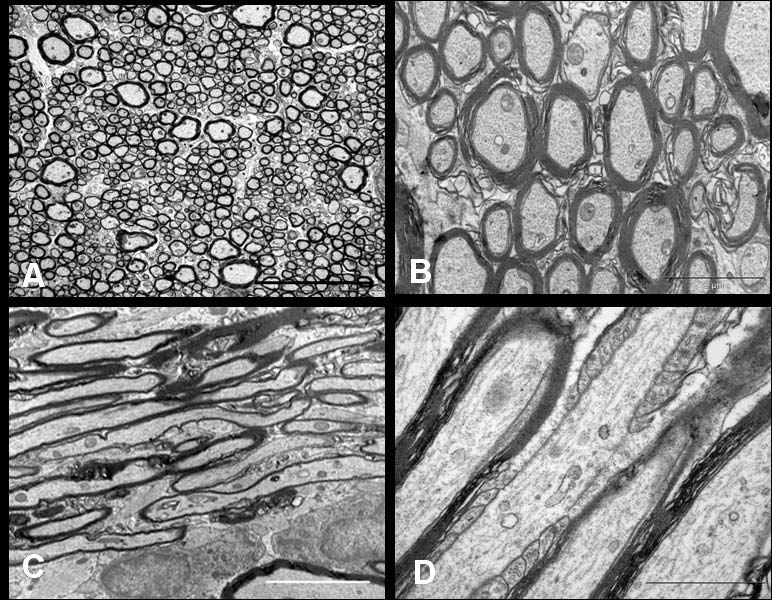
**EM of the retro-orbital distal segment of rat optic nerve of the saline injected control animal immediately after the injection**. Axoplasm of the myelinated axons contain numerous neurofilaments, microtubules, mitochondria and various other organelles. The transverse sections (A, Bar = 5 μm and B, Bar = 2 μm) show compact arrangement of the myelin lamellae around the axons in the internodal regions. The longitudinal sections show parallel running myelinated axons (C, Bar = 5 μm). Axon-myelin relationship in the nodal-paranodal region is better appreciated at very high magnification (D, Bar = 1 μm) Here, myelin terminal loops are seen attached to the paranodal axolemma on either side of the node.

The retro-orbital optic nerve from saline injected animals at all time points displayed ultrastructural features similar to those previously described in the normal adult rat [26-29]. In transverse sections, well-fixed myelinated axons of various diameters maintained round to ovoid sectional profiles (Figure 7a) with numerous microtubules and neurofilaments dispersed evenly in the axoplasm. Microtubules were seen as hollow round cross-sections and neurofilaments as small electron-dense dots with no central clearing. Also seen in the axoplasm were mitochondria with normal morphology and intact cristae. Surrounding the axons, myelin remained compact with a normal periodicity with no intramyelinic lacunae or vacuoles. In longitudinal sections (Figure 7b), axons ensheathed by darkly stained myelin contained filamentous structures (neurofilaments and microtubules) which showed linear orientation, parallel to the length of the axons. The nodes of Ranvier displayed a normal morphology with well preserved paranodal terminal loops contacting axolemma and a non-myelinated nodal gap measuring less than or approximately equal to 1 μm. Various glial cells surrounded the axons. Oligodendroglial cells had an electron-dense cytoplasm and heterochromatic nuclei. Astrocytes were identified by the electron-lucent cytoplasm and processes which contained bundles of intermediate filaments. Microglia had heterochromatic nuclei similar to oligodendroglia, but their cytoplasm appeared less dense.

### Ultrastructural changes in NMDA-injected optic nerve axons

Optic nerves from NMDA-injected eyes, examined immediately and 24 hrs after injection were similar to the optic nerve of saline-injected eyes. At 72 hrs post-NMDA insult, unmyelinated fibres running in the INFL maintained normal morphology; however, pathology was identified in the retro-orbital optic nerve. After careful observation of cross sections of proximal and distal segments, three distinct abnormalities were identified with changes appearing more pronounced in the distal as compared to the proximal optic nerve (data not quantified). These ultrastructural changes had similar spatiotemporal and pathological features to that described in classical Wallerian degeneration [30,31].

#### 1) Axonal swellings

**Figure 8:**
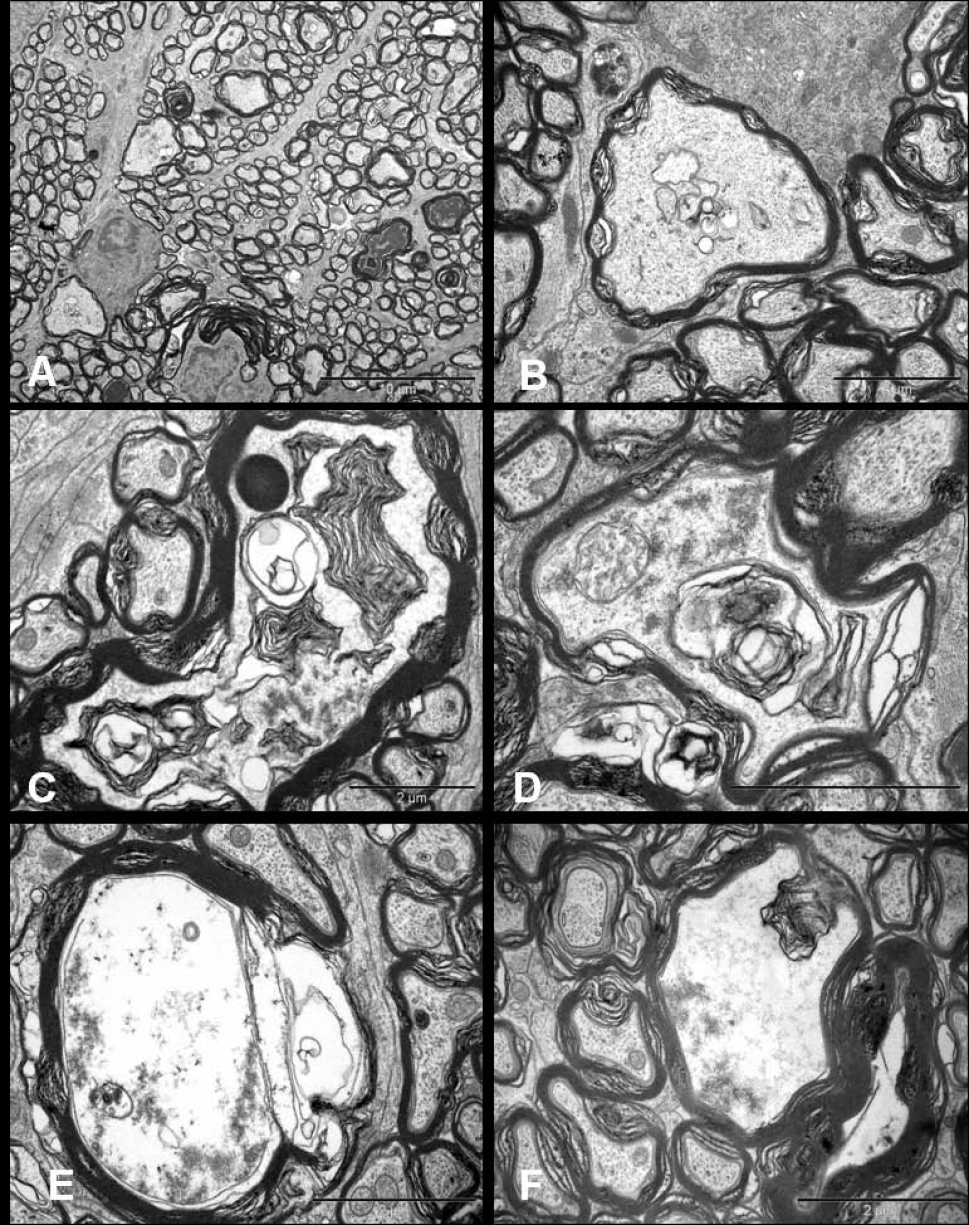
**Ultrastructural appearances of axonal swellings in the transverse sections of distal segment of rat optic nerve after 72 hrs of NMDA injection**. The major change observed is the appearance of swollen axons (A, Bar = 10 μm). The axoplasm of these axonal swellings show abnormal collection of altered tubulovesicular structures (B-D, Bars = 2 μm), cytoskeletal disintegration (C-F, Bars = 2 μm), and multilayered whorled masses (C & F, Bars = 2 μm), which are seen to be arising from the inner layers of the myelin (F, Bar = 2 μm).

Swollen axons appeared pale and enlarged with axolemmal expansion and cytoskeletal disintegration, characterizing 'watery degeneration'. The axoplasm was partially or completely devoid of organelles and cytoskeletal elements. Loss of microtubules with relative preservation of neurofilaments was observed in some axons. Many fibres contained dense accumulations of neurofilaments, altered tubulo-vesicular membranous organelles, mitochondria and multilayered whorled masses, which appeared to be arising from the inner layers of myelin. The myelin sheath surrounding these axons remained compact and unaltered at most places. (Figure 8)

#### 2) Dense axons

**Figure 9:**
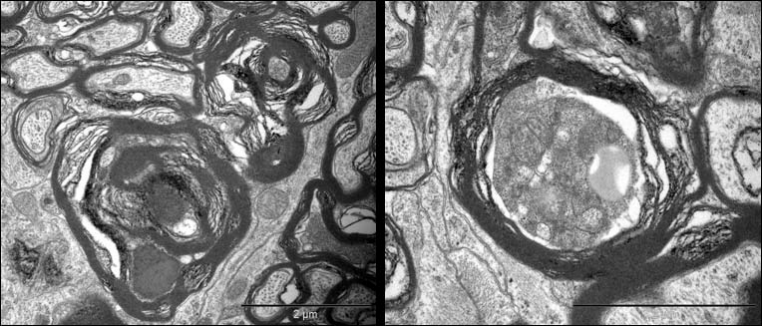
**Ultrastructural appearance of hyperdense axons in distal optic nerves seen 72 hrs after NMDA injection (Bars = 2 μm)**. Note that despite the extent of the changes, some adjacent axons still appear normal.

Some small to medium sized axons, which appeared dark under lower magnification, had their axoplasm filled with an amorphous, granular and dark material, thus portraying what is described as 'dark degeneration'. Although, organelles were visible in some fibres, it was difficult to define the composition of dense axoplasmic material even at very high magnifications. The myelin of dark axons did not show significant alterations. (Figure 9)

#### 3) Demyelination

**Figure 10:**
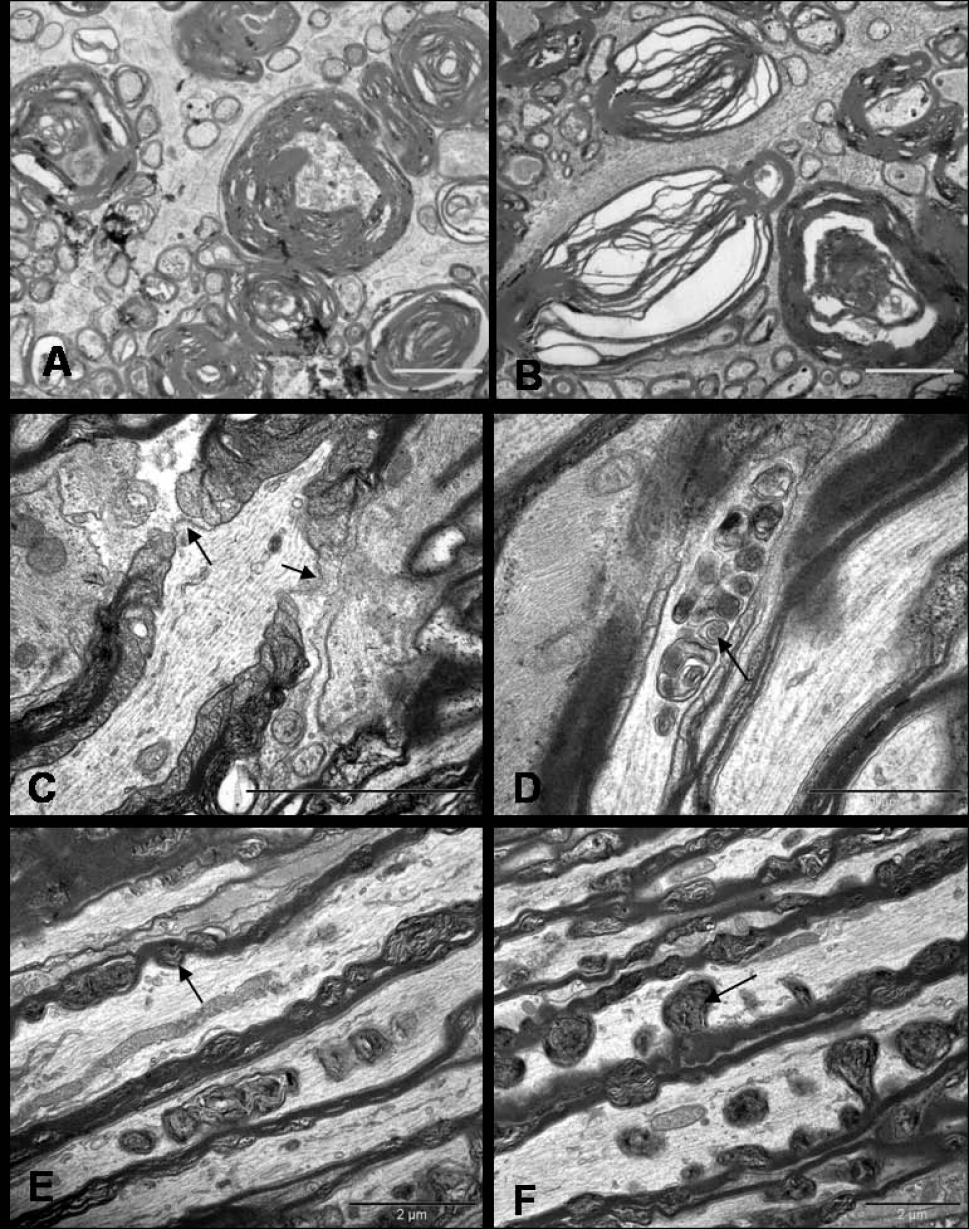
**Ultrastructure appearances of distal segment of rat optic nerve after 72 hrs of NMDA injection**. Transverse sections show separation and vacuolation of myelin sheath (A & B). Longitudinal sections display nodal blebs (C, arrows), abnormal accumulation of altered organelles (D, arrow), myelin whorls (E, arrow) arising from the inner myelin and forming mesaxon (F, arrow). The cytoskeleton surrounding the myelin whorls appears normal at this stage (E & F). Bars = 2 μm.

Occasional fibres showed features of demyelination which included vacuolation and splitting of the myelin sheath. These demyelinating changes were mainly seen around abnormally dense/dark axons. Partial or complete loss of axon transformed the myelin into collapsed structures which appeared as 'myelin bodies' in the extracellular space. Few normal axons also showed myelin changes such as lamellar separation or widening which made the myelin look abnormally thick and dark. (Figure 10A &10B)

Longitudinal sections displayed abnormal focal swellings and dense axons scattered between numerous normal fibres. Magnified images revealed some pathological changes even in fibres which appeared healthy under lower magnifications. The main abnormal features were abnormal accumulation of tubulo-vesicular structures including organelles in the nodal and paranodal region, formation of nodal blebs, and intermittent myelin proliferation where the fibres showed splitting and proliferation of inner layers of myelin in the internode. The myelin proliferations formed whorls and loops, which protruded into the axon carrying the axolemmal covering around them. In some places, the proliferation was so pronounced that the mesaxonal loops occupied the entire diameter of axon. The axoplasm around the myelin whorls looked normal. The later finding was strictly restricted to the distal segments. No disturbance in the axon-myelin relationship was observed in the paranodal regions and the myelin terminal loops maintained normal relationships to the axons. No myelin debris was seen inside the astrocytic and microglial cells. (Figure 10)

**Figure 11:**
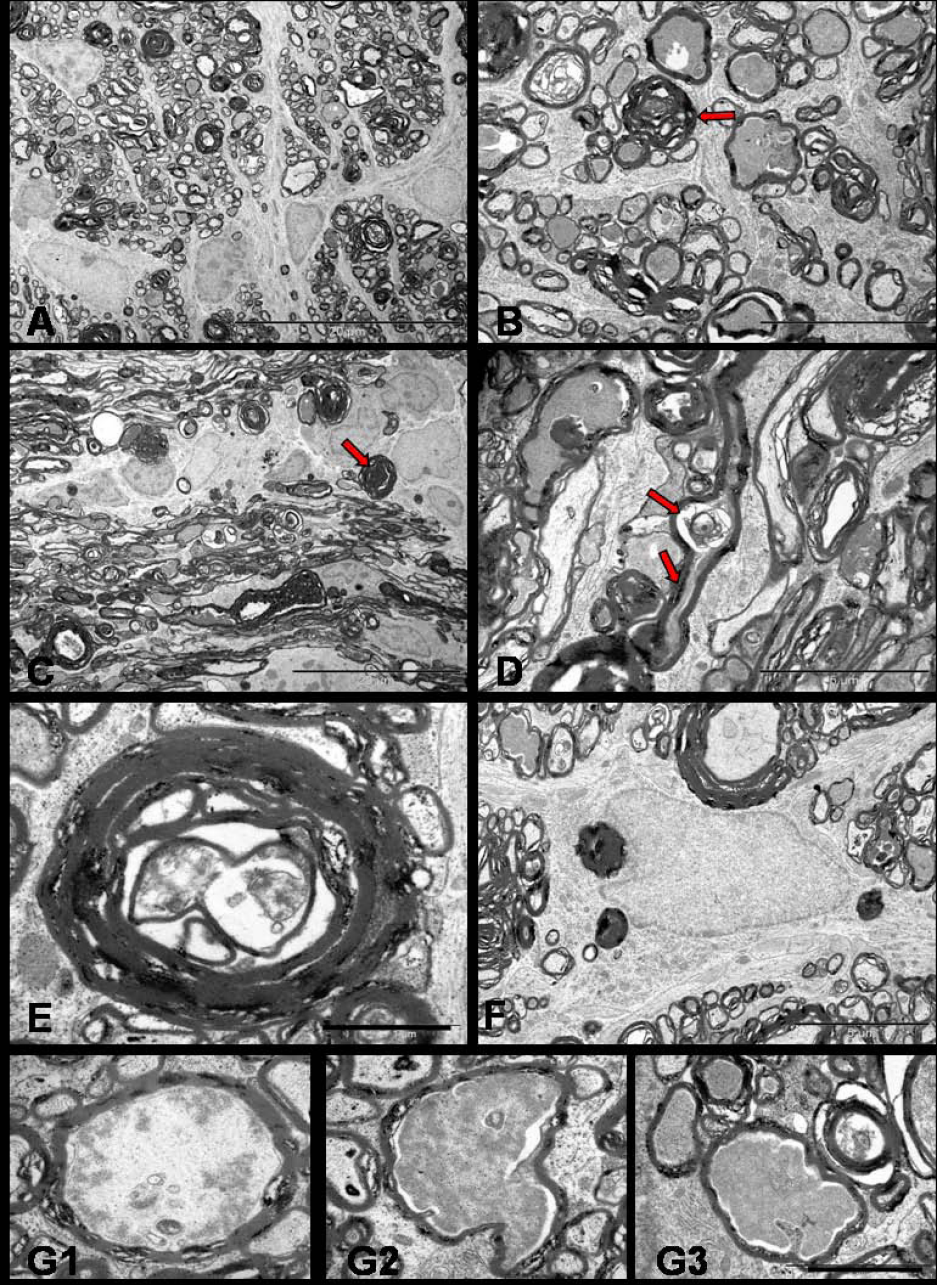
**TEM of distal segment of rat optic nerve after 7 days of NMDA injection**. Transverse (A, Bar = 20 μm & B, Bar = 5 μm) and longitudinal sections (C, Bar = 20 μm & D = Bar = 5 μm) show the fibres undergoing dark degeneration with most axons showing hyperdense axoplasm (red arrows). Longitudinal profile show the focal axonal swelling and hyperdense axoplasm in the same axon (D, arrows). Axon towards the end stage of degeneration (E, Bar = 1 μm) show nearly collapsed axon structure and the myelin debris phagocytosed by the astrocytes (F, Bar = 5 μm). Last series of photographs (G1, G2, G3, Bar = 2 μm) represent axoplasm in various stages of dissolution.

The degenerative changes at 7 days were clearly more intense than the previous stage. Extensive invasion by the filamentous astrocytic processes completely disorganized the nerve structure. Almost all fibres were altered and only a few scattered fibres showed a normal appearance. A remarkable feature at this stage was the predominance of dark fibres, as compared to watery fibres. These dark axons appeared shrunken on longitudinal sections to create a gap between the atrophic axon and the inner layers of myelin. Moreover, demyelinating changes such as myelin breakdown, detached and vacuolated lamellae and formation of myelin bodies were frequently seen. Phagocytosing cells including microglia, astrocytes and oligodendroglial cells were present throughout, and myelin debris was mostly an extracellular feature. The most striking finding on longitudinal sections, where a substantial length of axon was seen, was that the same axon showed features of watery degeneration (axonal swelling) and dark degeneration (hyperdense axoplasm). (Figure 11)

## Discussion

Most of the information about the pathology of axonal degeneration is derived from the experimental nerve transection model which causes classic Wallerian degeneration of axons [32] and a reactive gliosis [30,31]. Under the light microscope, NMDA induced excitotoxic injury to the retina causes significant reduction in thickness of inner retina at 72 hrs (posterior retina, p = 0.002 and peripheral retina, p = 0.012) with thickness reducing further to 68% and 76% in both regions compared to the control eyes at 7 days (p < 0.05) [21]. This implies loss of RGCs and their dendrites. Damage to RGC somata is characterized by a well-ordered sequence of organelle changes along with a dying-back-like degeneration of the axons (the optic nerve fibres) [21].

There is paucity of literature regarding degenerative changes in the optic nerve at the ultrastructural level. To our knowledge, this is the first study to report the pathological changes in the optic nerve at ultrastructural level after excitototxic retinal damage. To characterize the events leading to neuronal cell death after isolated injury to the perikaryon, this morphologic study in rats describes the time-dependent pathological sequelae in the RGCs and optic nerve after NMDA-induced retinal damage. TEM analysis showed that the effects of excitotoxic stimuli begin in the retina within 24 hrs where RGCs undergo progressive necrosis, and the optic nerve degeneration mimics classic Wallerian degeneration. Studies have shown that Wallerian degeneration mutation rescues axons but not cell bodies in a rat model of glaucoma and that axonal degeneration can be delayed for weeks in the presence of the slow Wallerian degeneration gene (WldS), suggesting that Wallerian degeneration is an active, regulated process [33]. Recent research suggests that the more long-lived, functionally related WldS protein, a variant of Nmnat1, substitutes for endogenous Nmnat2 loss after axon injury, which is actually considered to prevent spontaneous degeneration of healthy axon [34].

### NMDA-induced retinal changes

Previous studies have shown that the excitotoxic injury could lead to apoptotic, autophagic or necrotic cell death [35-42]. Evidence is also available that the excitotoxic injury can produce hybrid forms of cell death, existing on a continuum between the classically defined apoptosis and necrosis [38,39,43], and is likely to depend on the degree of insult and the sensitivity of the exposed neurones. Time-dependent studies of glutamate exposure to cultured neuronal populations showed that the excitotoxins induce early necrosis and delayed apoptosis [36,44]. There is also evidence that the necrotic neurons may completely recover to undergo apoptotic transformation later [44]. The current study is consistent with previously reported pathology [45,46].

Cell death, seen 72 hrs after excitotoxic insult, exhibited the essential features of necrosis characterized by progressive organelle swelling, cytolysis and karyolysis. RGCs showed mitochondrial swelling, dilated ER, dissolution of ribosomes in early stages and disintegration of cytoplasmic organelles, change in nuclear morphology and mild chromatin aggregation in advanced stages [43,47-50]. Because apoptosis requires functional mitochondria [51], the presence of swollen and disrupting mitochondria suggested that the event was non-apoptotic. In the presence of whorl-like multi-laminated 'myelin figures' or 'myelin-like bodies' [52] in severely damaged RGCs, and in the absence of highly specific features of apoptosis (heterochromatin segregation, nucleolar disintegration or apoptotic bodies) as well as autophagocytosis (presence of typical autophagosomes) [52-55], cellular events were labelled as necrotic.

### NMDA-induced optic nerve changes

In comparison to the popular models of immediate (axotomy) or delayed (stretch) disruptive injuries, where the axons and myelin are simultaneously and directly damaged at the site of lesion, optic nerve fibres in the current model do not suffer any form of direct injury. Because the optic nerve is physically isolated from the eyeball, retro-orbital axonal changes seen in the present study are most likely the result of direct injury to RGCs or indirect damage to the intraretinal axons. Several physiological studies suggest that the axons lack excitatory amino acid receptors and they respond to excitatory amino acids indirectly by the change in extracellular ion composition associated with neuronal depolarization [56-58].

The initial sequence of events resulting in axonal degeneration depends upon the type of injury. During early Wallerian degeneration, asymmetric paranodal myelin retraction was seen as the initial event after axotomy in frog optic nerve followed by the formation of nodal blebs and accumulation of abnormal organelles in nodal axolemma [59]. In response to excitotoxic perikaryal injury, this study found nodal changes in the form of bleb formation and abnormal accumulation of organelles in the paranodal region with no obvious myelin terminal loop retraction as early changes. These changes resembled the response observed after non-disruptive stretch injury, where accumulation of membranous organelles in the paranodal and internodal regions preceeded the nodal bleb formation related with loss of axolemmal undercoating [60].

Nodal changes seen in the present study indicate the role of disrupted ionic equilibrium in initiating axonal damage following excitotoxic perikaryal injury. It has been proposed that the decreased ATP and mitochondrial formation, usually seen with necrotic cell death, results in energy-dependent pump failure at active nodal sites causing ionic imbalance, focal cytoskeletal dissolution and neurofilament compaction; loss of membranous Ca^2+^-ATPase pump causing Ca^2+ ^influx induce calpain-mediated proteolysis of the subaxolemmal proteins which results in the formation of nodal blebs [61,62]. This proteolytic activity spreads to involve the entire nodal axoplasm [63] results in focal axonal swellings with variable amount of cytoskeletal disruption. Studies have also shown that proteolyic degradation of sidearms of neurofilaments results in their axoplasmic aggregation [64]. These cytoskeletal changes are likely to affect the axonal transport system leading to the accumulation of transport material including vesicles, organelles, proteins and enzymes in the paranodal and internodal regions [65].

Similar to the study using optic nerve crush injury, the current study also identified watery and dark degeneration in the axons [66]. Both patterns were observed at 72 hrs and 7 days of injury. Although there was no apparent predominant form at 72 hrs, there was a clear increment of fibres undergoing dark degeneration and demyelination at 7 days. It is unclear whether the same axon displays different type of axoplasmic degeneration at variable distance from RGC at the same time or individual axons undergoes a specific type of degeneration throughout its length. It is possible there is a cause and effect relationship between both types of degeneration, but evidence is circumstantial. Unlike the stretch injury model, axons form the only link between the myelin and the cell body in the current study. It was presumed that perikaryal insult is unlikely to damage myelin without producing axonal changes. However, myelin showed proliferation and intermittent separation at internodes in the absence of cytoskeletal damage.

Glial cells in the optic nerve also reacted to excitotoxic-induced axonal degeneration in a manner similar to that seen during Wallerian degeneration [67]. Although, there are evidences for the expression of NMDA receptors on oligodendrocyte processes in white matter [19], oligodendrocytes in retro-orbital optic nerve axons remained normal. But astrocytes underwent reactive changes with the development of extensive filament-rich processes. Studies have shown that astrocytes and microglial cells invade the myelin sheath at the intraperiod line and phagocytose the peeled off outer lamellae [68]. No such glial invasion was seen in this study. Myelin debris was seen scattered in the extracellular space. Phagocytosed myelin which is initially in the form of the paired electron-dense curvilinear lines decompose and form a homogeneous or heterogeneous osmophilic layered structure, the myelin body, which, in the final stages, disintegrate and transform into globoid lipid droplets and needle shaped cholesterol crystal [68].

## Conclusion

In conclusion, selective perikaryal excitotoxic injury causes a predominantly necrotic form of somal death with simultaneous nodal-paranodal changes in axons culminating later to Wallerian-like degeneration in the form of dark and watery degeneration with demyelination. The Wallerian-like degeneration noted in this model, after primary perikaryal injury, raises the possibility that excitotoxicity-induced axonopathy is an active, regulated event. This hypothesis could be tested by using the current model and comparing the axonal degeneration in slow Wallerian degeneration (WldS) rats with the degeneration in control rats.

## Authors' contributions

SS, the main author, prepared the study design after extensive literature review, collected the tissue samples, carried out electron microscopy and photography, analysed and interpreted the data. She also drafted the manuscript, made the changes as per supervisors and reviewers suggestions with the help of HC, who also assisted in collecting tissue samples, and made important intellectual contributions in the analysis of data. RC, being the principle supervisor, participated in study design, supervised each step of the study, made important suggestions on the recent updates on the research topic with contribution in reviewing the manuscript. PB, being an experienced neuropathologist, helped in thorough analysis of ultrastructure of optic nerve. All authors read and approved the final manuscript.
